# Editorial Note: Multiple Pathogens Including Potential New Species in Tick Vectors in Côte d’Ivoire

**DOI:** 10.1371/journal.pntd.0014341

**Published:** 2026-05-20

**Authors:** The PLOS Neglected Tropical Diseases Editors

The *PLOS Neglected Tropical Diseases* Editors issue this notice to update the previously published Expression of Concern on this article [1–3].

Following the publication of the article and Expression of Concern [1,3], PLOS investigated concerns pertaining to the reported ethical approval and the article’s adherence to *PLOS Neglected Tropical Diseases*’ research ethics policies.

Specifically, concerns were raised that the ethics statement of this article [1] states that the study was approved by the Côte d'Ivoire Ethics committee under agreement N°86/MSLS/CNERN-dkn, but the reported ethics approval document was also cited in [4, 5, retracted in 6] despite there appearing to be differences in the subjects, the samples collected, and the sample collection period reported in these articles.

PLOS received a copy of the ethics approval document N°86/MSLS/CNERN-dkn as part of the editorial investigation in [4–6]. This document was issued on October 31, 2014, by the Ministère de la Santé et de la Lutte contre le SIDA of the République de Côte d’Ivoire for a study titled “*Identification des agents pathogènes responsables de la fièvre dans la région de Yamoussoukro, République de Côte d’Ivoire*”.

PLOS reviewed the documentation provided by the institution and concluded that the documents did not fully resolve the journal’s concerns. Therefore, the Expression of Concern stands.
